# Modulatory Effects of *Arctostaphylos uva-urs* Extract In Ovo Injected into Broiler Embryos Contaminated by Aflatoxin B1

**DOI:** 10.3390/ani12162042

**Published:** 2022-08-11

**Authors:** Hamada Elwan, Abdelhameed S. A. Mohamed, Dawood Hosni Dawood, Shaaban S. Elnesr

**Affiliations:** 1 Animal and Poultry Production Department, Faculty of Agriculture, Minia University, El-Minya 61519, Egypt; 2 Agricultural Chemistry Department, Faculty of Agriculture, Mansoura University, Mansoura 35516, Egypt; 3 Department of Poultry Production, Faculty of Agriculture, Fayoum University, Fayoum 63514, Egypt

**Keywords:** broiler, embryotoxicity, aflatoxin B1, *Arctostaphylos uva-ursi*

## Abstract

**Simple Summary:**

The poultry industry is of great importance worldwide; however, mycotoxins contamination, especially aflatoxins, has harmful effects on poultry production. Therefore, finding out new strategies to alleviate aflatoxin contamination is an important trend of research. In our study, we focused on the in ovo injection techniques using the methanolic extract of brae beery “*Arctostaphylos uva-ursi*” (*Ar. uu*) to modulate the aflatoxinB1 (AFB1) adverse effects in broiler embryos. As a result, 0.1 g of *Ar. uu* in ovo injection alleviates AFB1 embryotoxicity by enhancing chicks’ physiological responses and optimizing antioxidant status, which is reflected in a low mortality rate and heavier relative weight of the hatched chicks. The in ovo injection of AFB1 + *Ar. uu* significantly declined AFB1-induced toxicity in embryos and increased broiler chicks embryo’s survival by 62.5%, increased relative embryo weight by ∼65.25% compared to eggs injected with AFB1 alone. Regarding immune responses, the in ovo injection of *Ar. uu* enhances the embryo’s humoral immune responses and regulates oxidative stress biomarkers. In general, the in ovo injection of *Ar. uu* modulates broiler chicks’ embryotoxicity caused by AFB1.

**Abstract:**

In ovo injection of nutrients can modulate the embryo’s physiological responses against aflatoxin B1 (AFB1) embryotoxicity. This hypothesis was tested using in ovo injection of *Arctostaphylos uva-ursi* (*Ar. uu.*) methanolic extract. The total polyphenols, total flavonoids, total antioxidant capacity, and GC-MS analysis were all assessed in the *Ar. uu.* methanolic extract. A total of 180 ten-day-old embryonated eggs were distributed into six groups of 30 replicates each. The first group was used as a control (non-injected), and the second, third, fourth, fifth, and sixth groups were injected with 10 µ double-distilled water (DDW), 500 µL methanol, 0.01 g *Ar. uu.*/500 µL methanol, 50 ng AFB1/10 µL DDW, and 50 ng AFB1 in 10 µ DDW + 0.01 g *Ar. uu.*/500 µL methanol, respectively. The relative embryo weight, residual yolk sac weight, tibia length and weight, and survival were recorded. Total and differential leukocytes, oxidative stress, and humoral immune responses were observed. The residual yolk sac was lower (*p* < 0.05) in the *Ar. uu.* group than other groups. The embryonic growth (tibia weight and length) was enhanced in AFB1 + *Ar. uu.*-injected embryos compared with those injected with AFB1 alone. In conclusion, in ovo injection *of Arctostaphylos uva-ursi* could modulate AFB1-induced toxicity in chicken embryos.

## 1. Introduction

Contamination of poultry feed with mycotoxins, particularly aflatoxin B1 (AFB1), is a major problem for the poultry business; aflatoxicosis causes economic losses attributable to inefficient feed utilization, reduced egg production, and greatly reduced body weight growth as well as higher mortality [1,2]. Mycotoxins are naturally occurring toxins generated by fungus in their surroundings. The most well-known mycotoxins are aflatoxins, which affect poultry health [3]. These poisons can infect birds without creating specific clinical signs. Therefore, they are not recognized, except when the birds are autopsied, i.e., after the loss occurs or death [4]. The fungi grow even during shipping, transportation, and storage in silos [5]. The types of fungal poisoning in poultry vary depending on the type of toxins and their concentration in the diet, including the acute phase, which occurs when birds are fed a high concentration of mycotoxins, or the chronic phase, which occurs when birds are fed low-mycotoxins diets [6]. Egg production has declined today with high mortality [7] and repeated pathogenic infections caused by low avian immunity [6]. Aflatoxin’s (AFs) contamination in the diet causes harmful effects on the liver, induces jaundice, and changes the color to a yellowish color [3]. Aflatoxins cause atrophy in the Fabricius gland, immunodeficiency, increased spleen and kidney weights, kidney failure, increased water consumption, bleeding in different body organs or under the skin, and oedema [8]. Aflatoxins have no antigen, so the body has no immuno-antibodies [9]. The level of aflatoxins in poultry feed ranged from 10 to 20 g/kg and could be transmitted to eggs produced between 1/2000 and 1/2500 [10]. In general, the estimated values of AFs in the bread diet are 50 g/kg of diet that can be transmitted to eggs and embryos, resulting in fetal mortality or decreased vitality and incubation [11]. However, little is known about the toxicity and detoxification processes of early embryonic cells. The chick embryo develops detoxification capabilities by the fifth to the sixth day of incubation, and shortly thereafter the kidneys and liver are functionally established [12,13,14,15]. 

Detoxification tools of AF-contaminated feed that are both feasible and cost-effective are in high demand. In addition to preventive maintenance, physical, chemical, and biological procedures have been used to disinfect aflatoxins (Afs) from contaminated feeds and feedstuffs [16,17]. The use of non-nutritive and adsorbent materials in the diet to attach AFs and lessen AFs adsorption from the digestive tract has been one approach to the problem [18,19,20,21]. Using herbal extracts containing essential oils is one of the tools for combating mycotoxicosis in poultry. Natural components of the diet, minerals, phytochemicals, and xenobiotics have all been shown to influence drug-metabolizing enzymes and AFB1-adduct development [22]. Phenolic phytochemicals are thought to promote optimum health in part by serving as antioxidants and free radical scavengers, shielding cellular components from free radical injury. However, their antioxidant capacities are likely to vary due to their distinct chemical compositions [23,24,25]. Basic oils are complex molecules with a broad variety of chemical compositions and quantities of different compounds [26]. Essential oils are classified into two classes, terpenes and phenylpropenes, based on the amount of 5-carbon building blocks in each. However, their lipophilic property and chemical composition has been speculated to play a crucial role [27]. Because of their lipophilicity, terpenoids and phenylpropenes could be able to infiltrate bacterial membranes and enter the inner part of the cell [28]. Furthermore, structural properties such as the presence of functional groups [29] and aromaticity [30] contribute to essential oils’ antibacterial activity. Herbs possess a high concentration of phenolic chemicals, which are natural antioxidants [31]. Bearberry, known as *Arctostaphylos uva-ursi* L. (*Ar. uu.*), is a member of the evergreen heath family. The astringent leaves have long been used to treat bladder infections and other infections of the urinary system [32]. The antioxidant effects of *Ar. uu.* are most likely due to the glycoside arbutin fraction [33,34]. Dykes et al. [35] reported that *Ar. uu.* extract has antibacterial properties against microorganisms found in food. The glycosides arbutin (5–15%) and methyl-arbutin (up to 4%), as well as small amounts of free aglycones, are the most important components of *Ar. uu.* leaves [36]. Other ingredients include gallic acid, syringic acid, tannic acid, Gallo-tannins, galloylarbutin, *p*-coumaric acid, ursolic acid, and flavonoids, including glycosides of quercetin, myricetin, and kaempferol [37]. *Arctostaphylos uva-ursi* is a rich source of natural antioxidants. However, the effects of *Ar. uu.* fruit methanolic extract against AFB1 embryotoxicity has not yet been reported. Thus, in this study, we determine the efficacy of methanolic extract of *Arctostaphylos uva-ursi* fruit in protecting chicken embryos from AFB1 toxicity by in ovo injection of AFB1 with or without *Arctostaphylos uva-ursi* fruit methanolic extract into embryonated eggs.

## 2. Materials and Methods 

The experimental technique used in this study followed Egyptian animal welfare requirements and was approved by the Animal Care and Use Committee of Minia University, Faculty of Agriculture, Department of Animal and Poultry Production (APRIL24-2021), El-Minya, Egypt.

### 2.1. Arctostaphylos uva-ursi In Vitro Analysis 

#### 2.1.1. Preparation and Extraction of Methanol Extract

Fifty grams of *Arctostaphylos uva-ursi* were dried at 40–60 °C for 12 h and then soaked in MeOH at room temperature for 24 h. After filtration through filter paper (Whatman No. 1), the residue was re-extracted three times with MeOH under the same conditions, and to obtain a crude methanolic extract, the solvent was evaporated in a rotary evaporator at 40 °C [38].

#### 2.1.2. Determination of Total Phenolic Content

Total phenolic content was predicted spectrophotometrically using the Folin-Ciocalteau reagent, as previously mentioned by Limmongkon et al. [39]. The reaction mixture contained 0.5 mL of methanolic extract, Folin reagent (0.1 mL), and 7.5% Na_2_CO_3_ solution (0.5 mL). The absorbance at 740 nm was measured after 1 h of incubation at 25 °C in the dark. Each trial was carried out in triplicate. The phenolic content was calculated using an external gallic acid curve as a standard, and the results were expressed as mg gallic acid equivalent per gram dry weight (mg GAE/g DW).

#### 2.1.3. Determination of Total Flavonoid Content

The aluminum chloride colorimetric method previously stated by Munhoz et al. [40] was modified for total flavonoid content determination. Briefly, 2 mL MeOH, 0.3 mL of 10% AlCl_3_·6H_2_O solution, 0.2 mL of 1 M potassium acetate, and finally 2 mL of distilled water were added to 0.5 mL of MeOH extract. The absorbance at 430 nm was measured after 30 min of incubation at room temperature. Each trial was performed in triplicate. The measurement was calibrated using a standard curve of the prepared quercetin dihydrate solution, and the results were expressed in mg quercetin equivalent per gram dry weight (mg QE/g DW).

#### 2.1.4. Estimation of In Vitro Antioxidant Activity

The antioxidant activity assessment of methanol extract of *Arctostaphylos uva-ursi* was determined in vitro using the DPPH radical-scavenging assay. The assay was performed in triplicates and average values were considered. The free radical scavenging ability of the methanol extract against 2,2-biphenyl1-picrylhydrazyl (DPPH) was evaluated using the method declared by Dawood et al. [41]. In brief, a 0.1 mM DPPH solution in ethanol was prepared, and 3.8 mL of this solution was mixed with 0.2 mL of the methanol extract. The mixture was shaken and incubated at 25 °C for 60 min in the dark before measuring absorbance at 517 nm against a blank (water instead of samples and DPPH solution). The reaction mixture’s lower absorbance indicates greater free-radical-scavenging activity. The following equation was used to calculate the percent scavenging:

Inhibition (%) = (A blank − A sample)/(A blank) × 100(1)

where A sample is the absorbance of the test sample mixed with DPPH solution. A blank is the absorbance of the DPPH solution without a sample. 

#### 2.1.5. GC-MS Analysis

Gas chromatography (Agilent 7000C GC/MS Triple Quad, Santa Clara, CA, USA) equipped with an HP-5MS column (length = 30 m; diameter = 0.25 mm; film thickness = 0.25 m) mass spectrometer programmed at temperature 30–280/300 °C with a hold time of 5 min and rate of 10 °C/min was used to investigate bioactive compounds in methanol extract of dried *Arctostaphylos uva-ursi*. The chromatography conditions were as follows: column flow rate of 1 mL/min, injection mode split, and carrier gas Helium 99.999%. GC-MS spectra with mass library search (National Institute of Standards and Technology, Bureau Drive Gaithersburg, MD, USA, based AMDIS software) and relative retention indices were used to identify the components [42].

### 2.2. Arctostaphylos uva-ursi In Vivo Analysis

#### 2.2.1. Eggs Incubation Protocol 

Fertile broiler (Ross 308) eggs were obtained from a commercial breeder flock aged 38 weeks of age. Eggs were incubated in an incubator (Capacity 1500 eggs, Model JF-300, fully automatic incubator, El-Dahshan, Equipment Co., Ltd., Cairo, Egypt) under optimal incubation temperature (37.8 °C) and 65% relative humidity, and automatically turned every 2 h; then, after 10 days of incubation the eggs were candled with a lamp, and those containing dead embryos were removed from the incubator. The live embryos (*n* = 180) were then injected or not injected with 50 ng AFB1/10 µL or/and 500 µL of 0.1 g *Ar. uu.* fruit methanolic extract. On day 21 of incubation (hatching day), all rested eggs/groups were broken, and samples of residual yolk and tissues (liver, kidney, and heart) were taken. 

#### 2.2.2. Experimental Design

**Figure 1:**
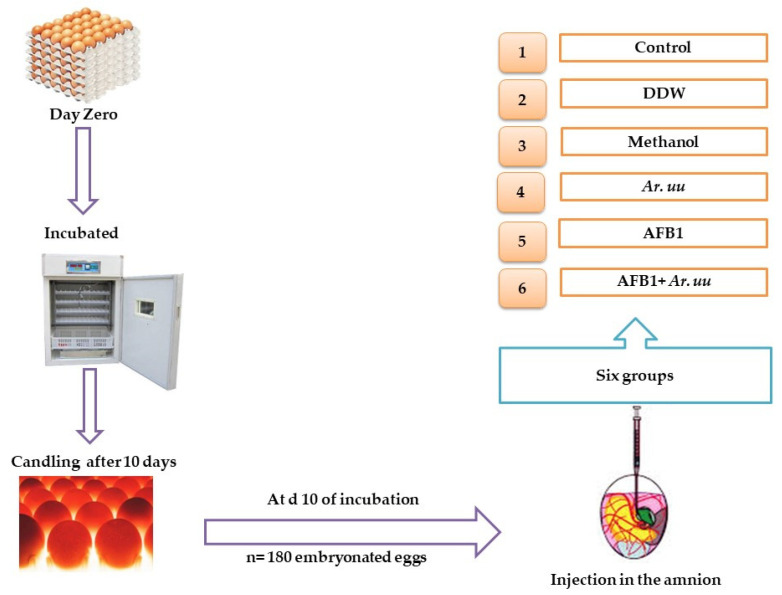
Experimental design where 1 = the 1st group served as a control (non-injected eggs), 2 = the 2nd group, only injected with 10 μL DDW, 3 = the 3rd group, injected with 500 µL Methanol, 4 = the 4th group, injected with 500 µL of a freshly prepared solution of 0.01 g of *Ar. uu.*, 5 = the 5th group, injected with 10 μL Methanol 20%, containing 50 ng AFB1 (AFB1 group), and 6 = the 6th group, injected with 50 ng AFB1 + 500 µL of 0.01 g *Ar. uu*.

Pure Aflatoxin B1 (AFB1) brushed from Sangon Biotech, Shanghai Co., Ltd. (Shanghai, China), (98% AFB1, catalogue no. A606874-0005) was dissolved with methanol (20%) to obtain final concentrations of 50 ng AFB1/10 μL. The AFB1 level was determined using an AFB1 ELISA Kit, product number: MM-1911O1, Romer Labs, Union, CN. At day 10 of incubation, eggs (*n* = 180) 58.6601 ± 2.6105 were randomly divided into six groups (30 eggs/group) using the YMC scale (METTLER instruments, YMC, Co., Ltd., Tokyo, Japan). A 21-gauge needle with a small, beveled tip was used to inject the eggs into the air cell, reaching for the amnion. Pilot testing using a visible dye before the injection indicated the solutions were safely delivered into the amnion. The 1st group served as a control (non-injected eggs), the 2nd group was only injected with 10 μL double-distilled water (DDW), the 3rd group was injected with 500 µL Methanol, the 4th group was injected with 500 µL of a freshly prepared solution of 0.01 g of *Ar. uu.*, the 5th group was injected with 10 μL Methanol 20 containing 50 ng AFB1 (AFB1 group), and the 6th group was injected with 50 ng AFB1 + 500 µL of 0.01 g *Ar. uu* (Figure 1). 

#### 2.2.3. Embryonic Development Indices 

On the day of hatch, the hatchability percentage and bodyweight of chicks were recorded. 

##### Effect of *Arctostaphylos uva-ursi* Fruit Methanolic Extract and/or AFB1 In Ovo Injection on the Growth Rate of Broiler Chicks 

On day 21 of incubation (hatching day), the hatched embryos from each group were individually weighed. Then relative yolk sac weight and mean relative embryo weight of each group were calculated.

##### Effect of *Arctostaphylos uva-ursi* Fruit Methanolic Extract and/or AFB1 In Ovo Injection on the Development of Tibia of Broiler Chicks

The tibia from each embryo at hatching day was removed, weighed, and expressed as relative to embryo weight. Tibia length was measured using a digital caliper.

##### Effect of *Arctostaphylos uva-ursi* Fruit Methanolic Extract and/or AFB1 In Ovo Injection on Serum Biochemical Indices and Antioxidant Biomarkers in the Serum and Tissues

At hatching time, fifteen blood samples from each treatment were randomly collected from the jugular vein before slaughtering and then centrifuged for 10 min (3000× *g*) at room temperature to separate the serum that was stored in Eppendorf tubes (1500 µL) at −80 °C until analyzed. Immunoglobulins A, M, and G were detected using ELISA kits according to the instructions of the kits (Nanjing Jiancheng Bioengineering Institute, Nanjing, China).

The tissue samples (liver, kidneys, and heart) were stored at −80 °C, then homogenized, after being diluted ten times (0.1 g per mL) using isotonic physiological saline; they were then centrifuged at 1295× *g* at 4 °C for 10 min, and the total protein content in the supernatant was determined using a total protein quantification kit. Then, the supernatant was collected for further analyses of glutathione (GSH), glutathione peroxidase (GSH-Px), total antioxidant capacity (TAOC), superoxide dismutase (SOD), malondialdehyde (MDA), and catalase (CAT) in tissues and serum in five samples, each group using Nanjing Jiancheng Bioengineering Institute (Nanjing, China) reagent kits.

##### Effect of *Arctostaphylos uva-ursi* Fruit Methanolic Extract and/or AFB1 In Ovo Injection on the Spleen Histological, Immunohistochemistry and Apoptosis Examination

The samples were fixed in 4% paraformaldehyde (PFA) and routinely processed in paraffin. Thin sections (5 μm) of each tissue were sliced and mounted on glass. Slides were stained with hematoxylin and eosin Y. Rabbit anti-AFB1 antibody was used in the immunohistochemical procedure. Apoptosis examination, using TUNEL assay techniques, was used as described in our previous work [43]. The tissues were observed and photographed with a digital camera (OPTIKA, B-383FL, Ponteranica, Italy). 

### 2.3. Statistical Analysis

Treatment-dependent alterations were analyzed using the general linear model approach (GLM). SAS version 9.4 (SAS Institute, Cary, NC, USA) was used for all analyses. The normality of distribution assumptions was checked using Shapiro Wilk followed by Levene’s test for homoscedasticity. Statistical differences among means were considered significant at *p* ≤ 0.05. A post-hoc test (Tukey–Kramer) was performed following ANOVA. Data are presented as means and pooled SEM. Graphpad prism 7 was used to draw graphs. 

## 3. Results

### 3.1. Arctostaphylos uva-ursi In Vitro Studies 

#### 3.1.1. Levels of Total Phenolic, Total Flavonoids, and Total Antioxidant Capacity 

The levels of total polyphenol, total flavonoids, and total antioxidant capacity of the methanolic extract of dried *Ar. uu*. were determined and illustrated in Table 1. The results exhibited that the phenolic content was 212.88 mg/g calculated as Gallic acid equivalent. Meanwhile, the total flavonoid content was 91.28 mg/g of Quercetin equivalent. In this study, we used the DPPH method to assess the antioxidant activity of an *Ar. uu.* methanol extract. The DPPH radicals were found to be inhibited by 60.25%.

#### 3.1.2. GC-MS Analysis

**Figure 2:**
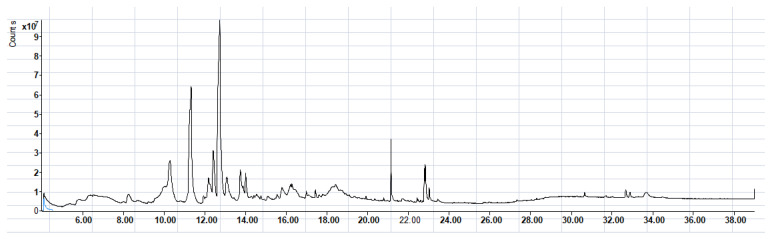
GC-MS Chromatographic profile of methanol extract of bearberry (*Arctostaphylos uva-ursi*).

To obtain information on the major metabolites present in the dried *Ar. uu.* fruit methanol extract, the GC-MS technique was used. More than 12 phytochemicals were found in the methanolic extract of *Ar. uu.* separated at different retention times (RT) (Figure 2 and Table 2). Based on the recorded results in Table 1, the chemical constituents obtained were classified as alkaloids, phenols, glycosides, coumarins, Cinnamic derivatives, and fatty acids. According to the % area, the analysis exposed the existence of 5-Hydroxymethylfurfural (43.83%), 4H-Pyran-4-one, and the major phytochemicals are 2,3-dihydro-3,5-dihydroxy-6-methyl- (24.45%), Thymine (17.40%), Benzofuran, 2,3-dihydro (11.34%), 2-Methoxy-4-vinyl phenol (0.75%), and Phloroglucinol, trimethylsilyl ether (0.55%). 

### 3.2. In Vivo Studies of Arctostaphylos uva-ursi Fruit Methanolic Extract 

#### 3.2.1. The Survival Rate of Broiler Embryos 

**Figure 3:**
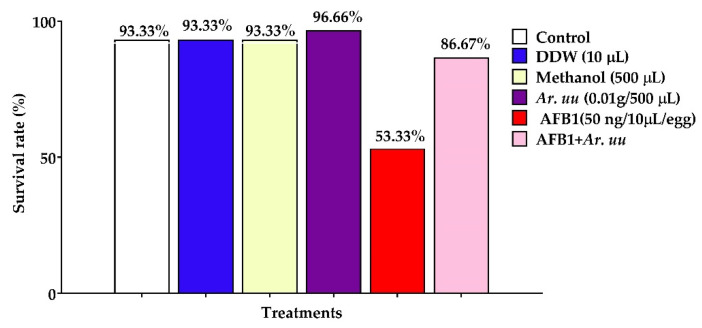
Effect of *Arctostaphylos uva-ursi* fruit methanolic extract and/or AFB1 in ovo injection on the survival rate of broiler embryos. Control = non-injected eggs, DDW = eggs injected with 10 μL DDW, Methanol = eggs injected with 500 µL Methanol, *Ar. uu.* = eggs injected with 500 µL of a freshly prepared solution of 0.01 g of *Ar. uu.*, AFB1 = eggs injected with 10 μL DDW containing 50 ng AFB1, and AFB1 + *Ar. uu.* = eggs injected with 10 μL DDW containing 50 ng AFB1 + 500 µL containing 0.01 g *Ar. uu*.

The effect of *Arctostaphylos uva-ursi* fruit methanolic extract and/or AFB1 in ovo injection on the survival rate of broiler chicks is illustrated in Figure 3, showing that the in ovo injection of *Ar. uu.* plus, AFB1 augmented the survival rate of broiler embryos by 62.51% compared with those injected with AFB1 alone. However, the in ovo injection of 500 µL of 0.1 g *Ar. uu.* into the broiler enhanced embryos’ survival rate by 3.56% compared to the control. Additionally, the in ovo injection of 50 ng AFB1 into the broiler embryos declined the survival rate by 39.64% compared with negative controls. There were no differences among negative controls (non-injected, DDW, and Methanol) in the survival rate.

#### 3.2.2. The Relative Residual Yolk Sac Weight and Relative Embryo Weight of Broiler Chicks 

**Figure 4:**
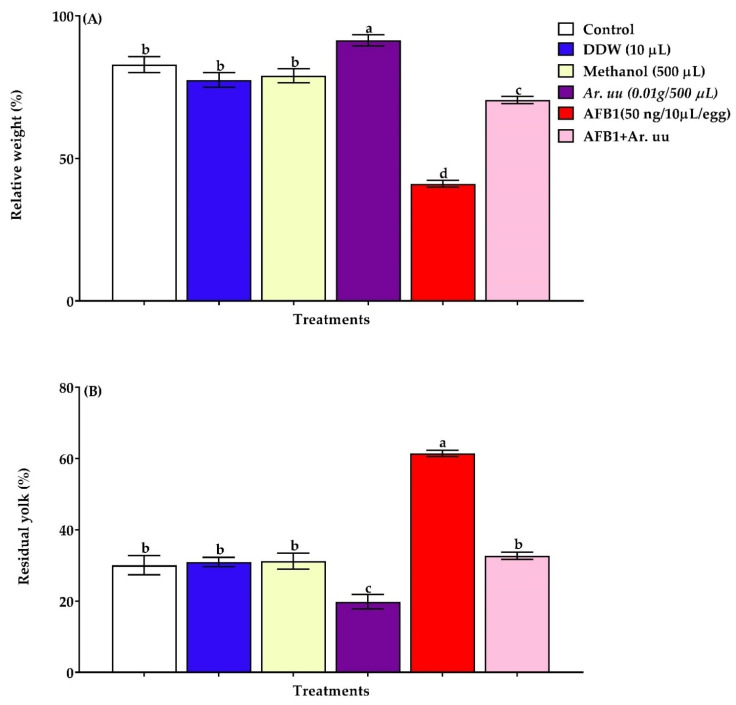
(**A**,**B**). Effect of *Arctostaphylos uva-ursi* fruit methanolic extract and/or AFB1 in ovo injection on checks relative weight and residual yolk relative weight of broiler chicks on 21 days of incubation (*n* = 15). Control = non-injected eggs, DDW = Eggs injected with 10 μL DDW, Methanol = eggs injected with 500 µL Methanol, *Ar. uu.* = Eggs injected with 500 µL of a freshly prepared solution of 0.01 g of *Ar. uu.*, AFB1 = Eggs injected with 10 μL DDW containing 50 ng AFB1, and AFB1 + *Ar. uu.* = Eggs injected with 10 μL DDW containing 50 ng AFB1 + 500 µL containing 0.01 g *Ar. uu*. ^a–d^ Values within columns with different letters significantly (*p* < 0.05) differ.

The relative yolk sac weight and relative embryo weight at hatching day (d21) were calculated as the percentage of the whole eggs each time, and the results of all groups are illustrated in Figure 4A,B. Data showed that the in ovo injection with *Ar. uu.* alone or with AFB1 increased (*p* = 0.01) embryos’ relative weights at hatching day by 29.34% compared to those in ovo injected with AFB1 alone; at the same time, 50 ng AFB1 in ovo injection had the lowest (*p* = 0.01) relative embryo weight (41.09%) and highest residual yolk sac relative weight (61.42%). Moreover, the relative residual yolk sac weight of embryos injected with AFB1 (50 ng/egg) was found to be augmented (*p* = 0.01) compared to other groups. In addition, 0.01 g *Ar. uu.* significantly lessened the relative residual yolk sac weight of embryos injected with AFB1 (50 ng/egg). Nevertheless, there were no differences in relative yolk sac weights among embryos in negative controls (AFB1 + *Ar. uu.*, DDW, and Methanol) and non-injected groups (control) (Figure 4B).

#### 3.2.3. Effect of *Arctostaphylos uva-ursi* Fruit Methanolic Extract or/and AFB1 In Ovo Injection on the Tibia Bone Development of Broiler Embryos 

**Figure 5:**
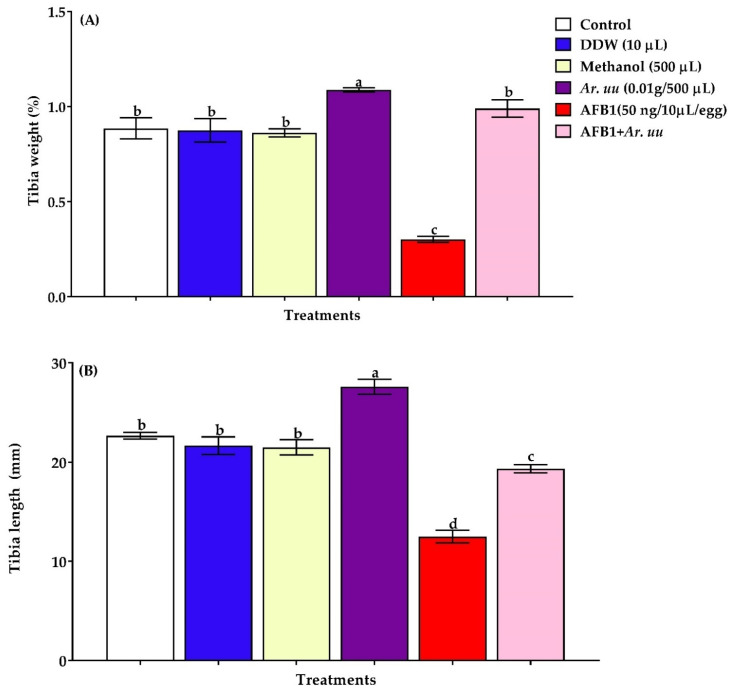
(**A**,**B**). Effect of *Arctostaphylos uva-ursi* fruit methanolic extract and/or AFB1 in ovo injection on checks relative tibia weight and tibia length of broiler chicks on 21 days of incubation (*n* = 15). Control = non-injected eggs, DDW = Eggs injected with 10 μL DDW, Methanol = eggs injected with 500 µL Methanol, *Ar. uu.* = Eggs injected with 500 µL of a freshly prepared solution of 0.01 g of *Ar. uu.*, AFB1 = eggs injected with 10 μL DDW containing 50 ng AFB1, and AFB1 + *Ar. uu.* = Eggs injected with 10 μL DDW containing 50 ng AFB1 + 500 µL containing 0.01 g *Ar. uu*. ^a–d^ Values within columns with different letters significantly (*p* < 0.05) differ.

The effect of *Arctostaphylos uva-ursi* fruit methanolic extract and/or AFB1 in ovo injection into broiler embryos on the tibia bone relative weight and length on 21 days of incubation are presented in Figure 5. Results showed that the in ovo injection of AFB1 alone decreased tibia bone relative weight and length at 21 days of incubation compared with other groups. However, the in ovo injection of *Ar. uu.* plus AFB1 increased tibia bone relative weight and length compared with those of the AFB1 group. Additionally, the in ovo injection with *Ar. uu.* alone augmented the tibia bone length and relative weight on the 21 days of incubation. However, there was no difference in tibia length and relative tibia weight among embryos in negative controls (DDW and Methanol) and the non-injected group (control) (Figure 5A,B).

#### 3.2.4. Liver and Kidneys Functions of Broiler Chicks 

The influence of AFB1 and/or *Ar. uu.* in ovo injection on serum total protein profiles, enzymatic activities of GOT, GPT, AKP, GGT, and creatinine kinase uric acid and urea in the serum of newly hatched chicks is illustrated in Table 3. The in ovo injection of AFB1 alone resulted in higher levels of serum albumin, a higher albumin/globulin ratio, and increased enzymatic activities of GGT, AKP, GPT, GOT, uric acid, urea nitrogen, and creatinine kinase activity by 57.05, 290.72, 106.15, 186.89, 60.90, 172.82, 84.54, 76.22, and 76.22%, respectively, compared to the control group. The injection with *Ar. uu.* (500 µL) plus AFB1 (50 ng/egg) alleviated AFB1-induced liver damage by significantly lowering albumin and the Alb/Glo ratio. However, the activity of GGT, AKP, GPT, GOT, levels of uric acid, urea nitrogen, and creatinine kinase activity were declined compared to AFB1 alone. Nevertheless, no significant difference in total protein profile, enzymatic activities of GOT, GPT, AKP, GGT, uric acid, and urea nitrogen, and creatinine kinase activity was observed among negative controls (DDW and Methanol) and the non-injected group.

#### 3.2.5. Serum Lipid Profiles

**Figure 6:**
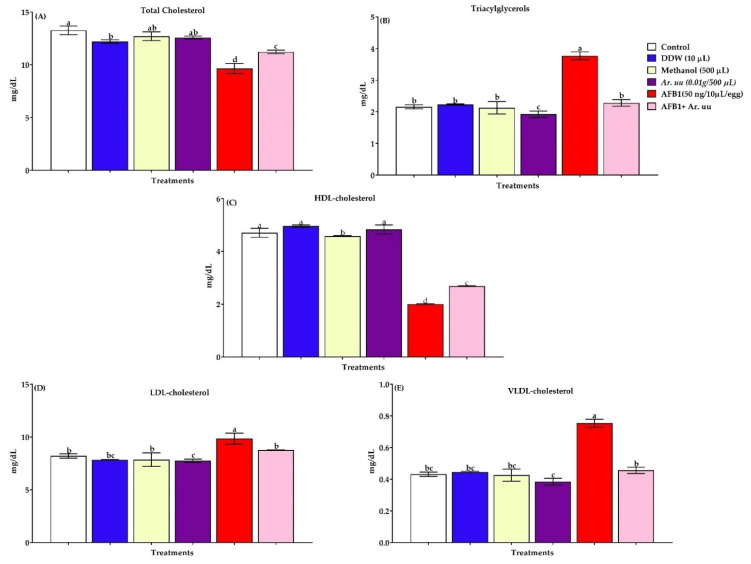
(**A**–**E**). Effect of *Arctostaphylos uva-ursi* fruit methanolic extract or/and AFB1 in ovo injection on lipid profiles of broiler chicks (*n* = 10). Control = non-injected eggs, DDW = Eggs injected with 10 μL DDW, Methanol = eggs injected with 500 µL Methanol, *Ar. uu.* = Eggs injected with 500 µL of a freshly prepared solution of 0.01 g of *Ar. uu.*, AFB1 = eggs injected with 10 μL DDW containing 50 ng AFB1, and AFB1 + *Ar. uu.* = Eggs injected with 10 μL DDW containing 50 ng AFB1 + 500 µL containing 0.01 g *Ar. uu*. ^a–d^ Values within columns with different letters significantly (*p* < 0.05) differ.

Serum lipid profiles (triacylglycerols, cholesterol, LDL-cholesterol, HDL-cholesterol, and VLDL-cholesterol) of new hatched broilers chicks exposed to AFB1 during incubation are displayed in Figure 6A–E. Results illustrated that AFB1 in ovo injection increased triacylglycerols and bad cholesterol indices (VLDL and LDL) compared to the control group. However, the injection of *Ar. uu.* recovered the adverse influences of AFB1 in ovo injection by reducing bad cholesterol levels and increasing HDL levels as good cholesterol. 

#### 3.2.6. Serum Thyroid Activity 

**Figure 7:**
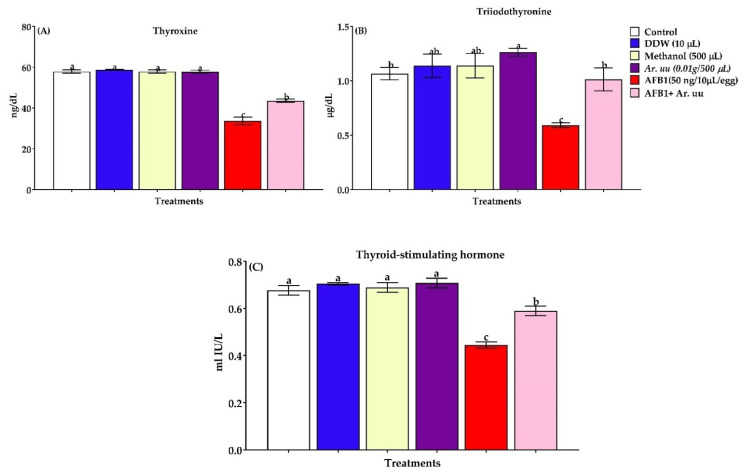
(**A**–**C**). Effect of *Arctostaphylos uva-ursi* fruit methanolic extract or/and AFB1 in ovo injection on thyroid function of broiler chicks (*n* = 10). Control = non-injected eggs, DDW = Eggs injected with 10 μL DDW, Methanol = eggs injected with 500 µL Methanol, *Ar. uu.* = Eggs injected with 500 µL of a freshly prepared solution of 0.01 g of *Ar. uu.*, AFB1 = eggs injected with 10 μL DDW containing 50 ng AFB1, and AFB1 + *Ar. uu.* = Eggs injected with 10 μL DDW containing 50 ng AFB1 + 500 µL containing 0.01 g *Ar. uu*. ^a–c^ Values within columns with different letters significantly (*p* < 0.05) differ.

Serum thyroid activity of newly hatched broilers chicks exposed to *Arctostaphylos uva-ursi* fruit methanolic extract and/or AFB1 during incubation is shown in Figure 7A–C. The changes in thyroxin, triiodothyronine, and thyroid-stimulating hormone indicated that AFB1 in ovo injection led to markedly decreased thyroid gland activity by 41.74, 44.33, and 34.41%, respectively, compared to the control group. However, the co-injection with *Ar. uu.* + AFB1 enhanced thyroid function by 29.19, 71.18, and 32.88%, respectively, compared with AFB1 alone. 

#### 3.2.7. Serum Total Immunoglobulins and Immunoglobulins Fractions

**Figure 8:**
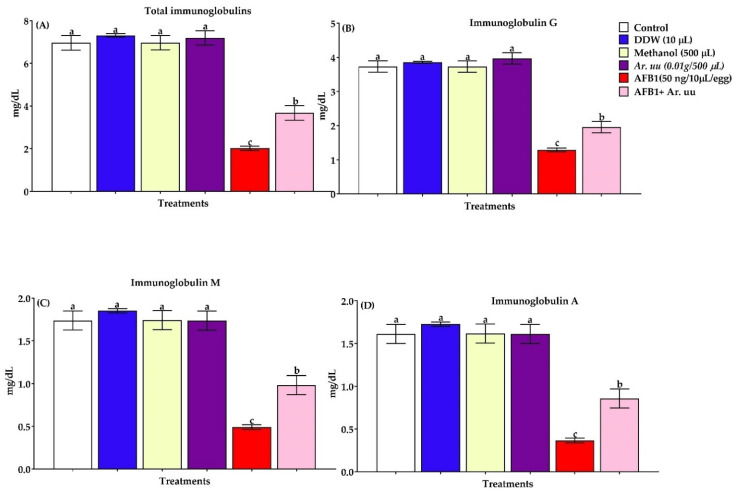
(**A**–**D**). Effect of *Arctostaphylos uva-ursi* fruit methanolic extract or/and AFB1 in ovo injection on serum total immunoglobulins, and immunoglobulins fractions of broiler chicks (*n* = 10). Control = non-injected eggs, DDW = Eggs injected with 10 μL DDW, Methanol = eggs injected with 500 µL Methanol, *Ar. uu.* = Eggs injected with 500 µL of a freshly prepared solution of 0.01 g of *Ar. uu.*, AFB1 = eggs injected with 10 μL DDW containing 50 ng AFB1, and AFB1 + *Ar. uu.* = Eggs injected with 10 μL DDW containing 50 ng AFB1 + 500 µL containing 0.01 g *Ar. uu*. ^a–c^ Values within columns with different letters significantly (*p* < 0.05) differ.

Total immunoglobulins and immunoglobulins fractions determined in the serum were shown in Figure 8A–D. Results indicated that the in ovo injection of AFB1 leads to downregulation of immunoglobulins and its fraction by 70.97, 65.41, 71.67, and 77.39%, respectively, compared with other groups. Contrarily, the in ovo injection of *Ar. uu.* + AFB1 recovered the harmful effects of AFB1 to be nearest the control group and better than the AFB1 group alone by 81.68, 51.16, 100.81, and 135.71% of total immunoglobulins, IgG, IgM, and IgA, respectively. Moreover, there were no significant differences among the control, DDW, Methanol and *Ar. uu.* groups. 

#### 3.2.8. Antioxidant Indices, Activities of Dehydrogenase Enzymes in Serum, Liver, Kidneys, and Heart Tissues of Newly Hatched Broilers Chicks

Data in Table 4 represent that compared with the control group, the in ovo injection of 50 ng AFB1 decreased (*p* = 0.01) total antioxidants capacity (TAOC) and the activities of enzymatic oxidative stress biomarkers (GSH-px, GSH, SOD, and CAT) in the serum by 55.96, 35.122, 33.76, 25.73, and 59.57, respectively, and the same trend was observed in tested tissues (liver, kidneys, and heart). Serum MDA had the opposite direction of other oxidative stress biomarkers, which was increased by 139.21%. The in ovo injection of *Ar. uu.* partially recovered the harmful influences of AFB1 by elevating the levels of TAOC, GSH, SOD, GSH-px, and CAT (75.56, 101.02, 15.78, 110.119, and 82.85%, respectively) and decreasing MDA activity by 45.75% compared with AFB1 alone. Concerning the activities of dehydrogenase enzymes (LDH, SDH, and GluDH), results revealed that the in ovo injection of 50 ng AFB1 augmented the activity of LDH and SDH by 71.86 and 273.96% compared with the control group and decreased the activity of GluDH by 34.32% in the serum, exhibiting the same trend in tissues. However, the in ovo injection of *Ar. uu.* plus, AFB1 leads to recovering the activity of dehydrogenase enzymes as partially to be nearest of the control. 

#### 3.2.9. Effect of *Arctostaphylos uva-ursi* Fruit Methanolic Extract and/or AFB1 In Ovo Injection on the Spleen Histology and Immunohistochemistry and Apoptosis Examination

**Figure 9:**
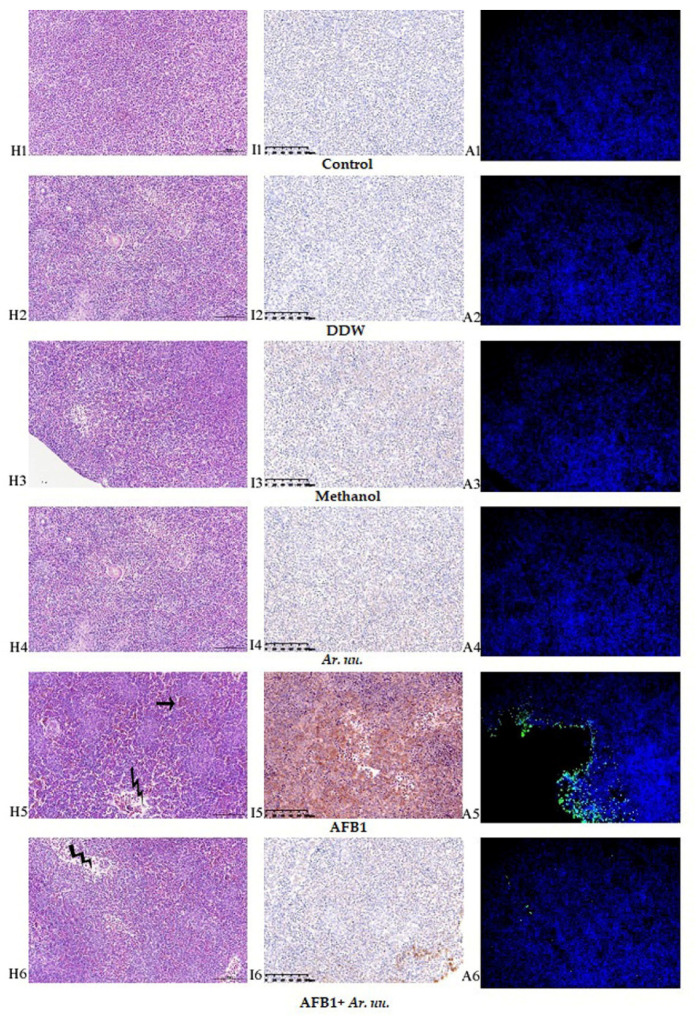
(**H1**–**A6**). Effect of *Arctostaphylos uva-ursi* fruit methanolic extract and/or AFB1 in ovo injection on the spleen histology (**H1**–**H6**) and immunohistochemistry (**I1**–**I6**) and spleen apoptosis examination (**A1**–**A6**). Control = non-injected eggs, DDW = Eggs injected with 10 μL DDW, Methanol = eggs injected with 500 µL Methanol, *Ar. uu.* = Eggs injected with 500 µL of a freshly prepared solution of 0.01 g of *Ar. uu.*, AFB1 = eggs injected with 10 μL DDW containing 50 ng AFB1, and AFB1 + *Ar. uu.* = Eggs injected with 10 μL DDW containing 50 ng AFB1 + 500 µL containing 0.01 g *Ar. uu*.

White and red pulps were identified in the chicks’ splenocytes. The splenic nodule, periarterial lymphoid tissue, and periellipsoidal lymphoid tissue were all split into the former. The chicken spleen contained ambiguous red and white pulp, dense periellipsoidal lymphoid tissue, and a few splenic nodules. In comparison to the control group, moderate congestion was found in select areas of the red pulp (Zigzag arrows), while lymphocyte density was mostly reduced (thin arrows) in the white pulp in the AFB1 group (Figure 9H1–H6). Immunohistochemistry Figure 9I1–I6 AFB1 groups had the highest AFB1 antibodies accumulation than the other groups, whereas the in ovo injection with *Ar. uu.* plus AFB1 (Figure 9I6) declined AFB1 antibodies accumulation nearest to controls. In comparison to the AFB1 group, the other groups had normal splenocytes. The TUNEL experiment revealed that the nuclei of TUNEL-positive cells were stained luminous green at varied rates in all groups (Figure 9A1–A6). TUNEL-positive cells were seen in higher numbers in the AFB1 group than in the control group (Figure 9A5). Furthermore, when comparing the AFB1 group to the AFB1 + *Ar. uu* group, the TUNEL-positive cells in the AFB1 + *Ar. uu* group were lower than AFB1 alone (Figure 9A5). 

## 4. Discussion

Polyphenols are characteristically composed of flavonoids, phenolic acids, coumarins, and tannins [44]. Herbs’ biological properties have been linked to their ability to produce bioactive compounds with antioxidant and antimicrobial activity, with a particular emphasis on polyphenols that have both properties [45]. 

The total polyphenol results in our study appeared to differ from those detailed by Azman et al. [46], who stated that total polyphenol content in *Arctostaphylos uva-ursi* was 102.11 mg GAE/g DW, is nearly two-fold higher. Dragana et al. [47] discovered that the total flavonoid content in *Ar. uu.*, was 73.46 mg/g rutin equivalent, which was lower than the levels found in our study. The disparity between their findings and ours could be explained by different factors, including the regions and seasons in which the sample material was collected, the nature of phenolic components, extraction techniques, and the solubility of these compounds in different solvents with different polarities [48]. Commonly, the observed superiority of antioxidant activity is frequently attributed to the solubility of antioxidant compounds polyphenols and flavonoids, which are dependent on the polarity of the extraction solvent [49,50]. 

Aflatoxins cause aflatoxicosis in birds and lead to increased susceptibility to infectious diseases and the reduction of growth performance. The residual AFs in the egg can adversely affect hatchability, embryonic survival, and organ malfunctions [50]. Jelinek et al. [51] determined the embryotoxicity limits for AFB1 as 0.3–30 ng/egg and the teratogenicity limits as 3–30 ng/egg. In our study, relatively high doses of AFB1 (50 ng/egg) were used since the limits are frequently exceeded. Furthermore, Yin et al. [52] stated a variety of results for AFB1 concentrations in chicken eggs. As a result, finding effective techniques to protect fertilized eggs against aflatoxicosis is critical for the chicken industry’s long-term viability and sustainability. Using a chicken embryo model with in ovo AFB1 injections, previous studies reported that the chicken embryo’s development was negatively affected in the presence of AFB1 (10 to 100 ng/egg) [18,53]. Therefore, the current study investigated the efficacy of 500 µL of 0.1 g *Ar. uu.* in protecting chicken embryos from AFB1 toxicity, and the results clarified that the dose of AFB1 (50 ng/egg) caused significant embryonic mortality at hatching day, resulting in 44.67% mortality. Additionally, the presence of 500 µL of *Ar. uu.*, significantly reduced the mortality rate to 13.33% when embryos were injected with AFB1 (50 ng/egg). One of the major effects of aflatoxicosis on birds is a decrease in bodyweight, which directly affects the success of the poultry sector. Aflatoxin B1 inhibits the synthesis of DNA and RNA, thereby consequently decreasing the synthesis of protein that ultimately lessens the growth [54]. In the current study, in ovo injection of 500 µL of *Ar. uu.*, enhanced the relative embryo weight despite exposure to AFB1, signifying the potential protective impact of *Ar. uu.* to AFB1-injected embryos. Moreover, the residual yolk supplies more than 90% of the total energy requirements of the embryo via yolk lipids oxidation [55]. Yolk content is necessary for supporting embryo development during embryogenesis [56]. In the current study, the group injected with AFB1 had a significant increase in relative yolk sac weight and a reduction in the relative embryo weights. AFB1 has been reported to inhibit the growth and development of bone tissue in chickens, leading to the retardation of the development of the skeleton system, especially the tibia [57,58]. Chaudhry [59] reported that the length of the tibia and femur and the weight of the tibia, femur, ulna, and radius were significantly lowered in birds fed continuously with AFs (5 mg/g in feed) for 6 weeks compared with birds that did not receive AFs. In the present study, AFB1 (50 ng/egg) significantly declined tibia length and relative tibia weight compared to the other groups. In ovo injection of 500 µL of 0.1 g *Ar. uu.*, in the presence of 50 ng AFB1/egg significantly improved the tibia length as compared with that of embryos treated with AFB1 alone; this might be related to normal embryonic development due to nutritional active constituents in *Ar. uu.*

The current study demonstrated that the mortality rates were augmented in the groups treated with AFB1 compared to the control group. The hatchability rate in this study was decreased in the groups treated with AFB1. Khan et al. [60] determined that in ovo administration of AFB1 by embryo results in significant mortalities, embryonic malformations, and production of chicks with deficient immune systems. Aydin et al. [61] reported that in ovo administered aflatoxin Bl declined hatching weight in a dose-dependent manner. Oznurlu et al. [17] illustrated that in ovo administered AFB1 negatively affected the embryonic growth and development of the bone tissue. Thus, the results exhibited that *Ar. uu.* injection reduced the AFB1 negative effects on the development of the embryo. 

Bioactive compounds derived from medicinal plants, such as phenolic, anthocyanin, and flavonoid chemicals, have been employed as alternative therapeutic tools for treating different ailments throughout history [62]. Numerous vegetables are supposed to be able to intercept and inhibit free radicals, such as hydroxyl radicals (OH), hydrogen peroxide (H_2_O_2_), and superoxide anion radicals (O_2_), which cause oxidative injury in biomolecules due to secondary metabolites with antioxidant properties [63]. Furthermore, bioactive molecules derived from plants are recommended over synthetic antioxidants due to their superior safety characteristics [64]. As a result, there is an increasing interest in discovering natural substances that might reduce oxidative damage, which is at the root of many illnesses’ etiology. Bearberry has a historic medicinal usage against a variety of ailments, as it contains phytochemicals such as phenolic acids, flavonoids, and vitamins that are regarded to be the trigger bioactive that inhibit the inflammatory response, peroxidation, and the onset of a variety of noncommunicable diseases [65]. The modulatory effects in our study may be related to *Ar. uu.*, active compounds which are almost characterized as phenols, polyphenols, or phenolic acids. Our results show the TFC, TPC, and antioxidative activity of *Ar. uu.*, were 91.28, 212.88, and 60.253, respectively. Five-Hydroxymethylfurfural (5-HMF) was the most active constituent (43.83%) in our study. Recently, 5-HMF was found to have antioxidant activity by scavenging ABTS and DPPH free radicals [48]. According to the literature, compounds with Benzofuran (11.34%) structure have a wide range of therapeutic uses such as anti-inflammatory, antifungal, antibacterial, antidepressant, antitumor, antidiabetic, antioxidant, and others [66]. Hexadecanoic acid (0.43%) has also been shown to have anti-inflammatory, antimicrobial, and antioxidant properties [67]. Also, *P*-coumaric acid is a member of the hydroxycinnamic acid group. Polyphenols’ positive benefits are mostly attributed to their ability to alleviate the oxidative stress conditions that accompany these illnesses. Several polyphenols have been shown to have strong antioxidant activities in vitro since they can function as chain breakers or radical scavengers depending on their chemical structures [66,67]. Polyphenols have been shown to inhibit a variety of enzymes, including xanthine oxidase, telomerase, angiotensin-converting enzyme, lipoxygenases, metalloproteinase, cyclooxygenases, and protein kinases [68]; utilize the modulatory steps pathway [69]; be associated with intracellular receptors [70]; interact with the cellular cyclin-dependent regulation [71]; increase the synthesis of vasodilating molecules such as NO [72]; affect the platelet function [73]; possibly trigger detoxifying enzymes [74]; and interfere with caspase-dependent pathways [75]. Polyphenols perform their protective benefits primarily due to these qualities, and they are increasingly being studied as therapeutic agents for cancer and cardiovascular illnesses [74]. They may also provide indirect protection by stimulating endogenous defense mechanisms, according to research. Both GSH-px and glutathione S-transferase (GST) activities can reduce total intracellular GSH levels over time. GSH is conjugated with different electrophiles during GST-mediated processes, and the GSH adducts are actively released by the cell. In addition to their bioactivity, flavonoids demonstrated antiapoptotic, cell cycle progression and programmed cell death, cellular signaling alterations, and the control of immunological response [76]; the chemo-preventive action from several polyphenolic compounds might be due to the encouragement of both the intracellular antioxidant defense system and detoxifying activities [77]. Aflatoxin B1 is thought to be the most common and dangerous. Its carcinogenicity and immunosuppressive potential in all types of animals, including poultry, have been extensively described [22]. During AFB1 metabolism, aflatoxin B1-8,9-epoxide is the main metabolite produced in the liver by the biotransformation of AFB1 by mammals and birds’ cytochrome P450 enzymes. The nucleophilic binding of AFBO with glutathione to generate aflatoxin 8,9-dihydro-8-(S-glutathionyl)-9-hydroxyaflatoxin B1 is the principal detoxication process that inhibits DNA adduct formation (AFB1-GSH) [78]. The latest research has shown that numerous phytochemicals and flavonoids, such as quercetin, kaempferol, luteolin, and others, affect glutathione-related gene expression in colon tumor cells or breast cancer cells, specifically reducing GST expression. Recent research has found consistent structure–function correlations in which the structure can alter bioavailability, antioxidant capacity, and the ability to stimulate antioxidant/detoxifying enzymes [79,80,81]. A rising number of epidemiological studies have demonstrated that polyphenol consumption slows aging and aids in the prevention and treatment of cancer, neurological, myocardial, and neurological illnesses [82]. After accessing the animal body, the pathways through which polyphenolic compounds generate antioxidant activities would include four actions: increased oxidative enzyme activity, inhibition of lipid peroxidation, scavenging of reactive oxygen species in synergistic effects with some other nutrients [83], and decrease of oxidative stress via metal-ligand complexing [84]. These mechanisms are coupled to simulate the antioxidant capacity. The antioxidant action of phenolic content may very well be described as a hydrogen-atom transfer or a single redox reaction via protons [85]; nevertheless, catechins and flavonoids may also stimulate the generation of ROS in the body [86].

Concerning blood biochemistry, Kubena et al. [85] revealed that the liver is the target organ for the toxic effect of aflatoxins. Liver metabolism is disturbed by impaired conversion of enzymes, proteins, vitamins, amino acids, nucleic acids, and lipids [87]. In broiler chickens, the toxic effects of AFB1 are manifested by increasing activity of liver enzymes such as AKP, LDH GOT, GPT, and γGT and are used for evaluation of the severity of aflatoxicosis in broiler chickens [88]. Aflatoxins reduce the synthesis of protein, which may lead to decreased blood protein levels, causing a reduction in the efficiency of the immune system, as the important mechanisms of some immune responses are the factors produced that can kill pathogens, such as antimicrobial proteins and peptides [89]. The AFs intoxications have been stated as significantly reduinge levels of glucose, triglyceride, cholesterol, and total protein [90]. Aflatoxins are liposoluble components that are readily absorbed in the place of exposure (usually gut) into the bloodstream to the liver where they are metabolized in the microsomal system into detoxified or active metabolites [91]. AFB1 may occur as unconjugated or free forms of primary metabolites. Aflatoxins alter the absorption, synthesis, and transport of lipids to extra-hepatic tissues. Liver fatty acid composition is significantly changed among birds with aflatoxicosis [92]. AFB1-8,9-epoxide (formed by cytochrome P450 action on AFB1) significantly increased hepatic lipid peroxide levels. 

Thyroid hormones are necessary to maintain the systemic physiological balance in organisms [93]. Our results revealed that AFB1 lowered serum T3 and T4 concentrations. Nevertheless, these alterations were directly linked to TSH as described by the insignificant changes in TSH levels in AFB1-injected treatment compared with the controls. Low T3 and T4 level concentrations encourage T3 and T4 receptors in the thyroid gland, stimulating the release and synthesis of TSH [94]. Aflatoxins prompt lipid peroxidation in cells [95,96]. The damage to thyroid receptors has probably resulted from the aflatoxins-induced enhanced generation of reactive oxygen species, provoking lipid peroxidation. Low thyroid hormone concentrations indicate the development of metabolic disturbances. According to Berry and Larsen [97], aflatoxins inhibit 5-deiodinase, resulting in lower blood T3. The low levels of blood T3 and T4 could be attributed to the reduction of iodine concentrations, contributing to their synthesis [98]. The AFB1-caused damage to the gut epithelium reduces the dietary iodine absorption [99]. 

The intake of aflatoxin-contaminated diets leads to several undesirable influences in poultry; for instance, altered morphology of the liver [100], kidneys [101] and immune organs–thymus, bursa of Fabricius, and spleen [101], haematological and blood biochemical changes [101], and changes in thyroid hormones concentration can occur [93]. These results are linked to the resulting histopathological changes induced by AFB1 in the spleen, bursa, and thymus of exposed embryos and come in line with the resulting oxidative stress impacts of AFB1, where the antioxidant status has been associated with anti-inflammatory and immunosuppressive properties as stated by Lee [102].

The AFB1-induced immunotoxin influences have been well-recognized in the literature, including humoral response cell-mediated and innate immunity [103]. AFB1 has been shown to inhibit the development of the bursa of Fabricius and thymus [16], reduce the mitosis of B cells [104], decrease the weight of lymphoid organs [56], suppress the production of antibodies [105], and decline the phagocytic capacity and population of macrophages [95]. Aflatoxin’s intoxications suppress immunoglobulins (IgA, IgG and IgM) and augment the susceptibility of birds to bacterial, viral, and parasitic infections. At 0.5 to 1 mg/kg of aflatoxins, these interfere with T and B -lymphocytes functioning [106], atrophy of bursa of Fabricius [107], apparent change of splenic functioning, suppress the phagocytosis, cell-mediated immune response, and interferon production as well as complement system. 

AFs decline in serum proteins, owing to low α, β, and γ globulins, with IgG being more sensitive than IgM [106], may cause great suppression of acquired immunity from vaccination programs in certain disease models. The low levels of AFB1 appear to affect the vaccinal immunity negatively and may enhance the occurrence of diseases such as Marek’s disease, IBD virus, congenitally acquired salmonellosis and duodenal and cecal coccidiosis, etc., even in properly vaccinated flocks [9]. The failure of vaccines is associated with the immunotoxin impact of toxins that compromise for immune function of birds via prompting an inflammatory response and reducing cell-mediated immunity [101]. Reduction of the chemotactic ability of leucocytes, damaged heterophils, phagocytosis, and cellular and serum factors necessary for optimal phagocytosis can be detected in aflatoxicated birds [94]. 

The current findings clarified that injection of AFB1 significantly declined TIg, IgG, IgM, and IgA. The immunosuppressive influences of AFs owing to the direct inhibition of protein synthesis including IgA and IgG have been stated [108,109]. Additionally, AFB1 could boost the lysosomal digestion of IG. Moreover, it can decrease the production of lymphocytes by lymphoid tissues and diminish their ability to create cytokines. Injection of *Ar. uu.*, extract plus AFB1 into eggs boosted the formation of aflatoxin antibodies and lowered reprogrammed apoptotic cells. This might be due to *Ar. uu.*, extracts that contain active substances that work synergistically to inhibit the accumulative effects of AFB1. Aflatoxin B1 can prevent the migration of macrophages and intervention in the complement hemolytic activity [110].

Aflatoxins encourage the formation of free radicals and therefore cause liver peroxidation, leading to antioxidant depletion, apoptosis, and oxidative stress. All of these contribute to the development of malabsorption [88]. Aflatoxin B1 is the most biologically active form and causes liver lesions, immunosuppression, and poor performance in poultry [106]. It augments the production of free radicals, leading to lipid peroxidation and oxidative damage, which may eventually cause cell death and damage [89]. Eraslan et al. [101] investigated the influences of AFs on oxidative stress and detected a decrease in antioxidant activity in the erythrocytes of birds fed AFs compared to the control. Effects of Afs, especially AFB1, on antioxidant capacity signify the main problem for bird health. The current results illustrated that AFB1 significantly lessened the serum TOAC, GSH, GSH-px, and CAT while increasing MDA compared with the control group. In agreement with these results, AFs augmented MDA levels and declined antioxidant enzymes in chickens [105]. The increased production of ROS after AFB1 toxicity may be attributed to the AFB1 biotransformation to a highly reactive intermediate metabolite-AFB1 8,9-epoxide and producing free radicals causing oxidative damage [106]. Furthermore, ROS can react with the cell membrane and prompt its lipid peroxidation by allowing the lipid hydroperoxides to be progressively accumulated in the plasma membrane, which then decomposed to create MDA under stress or toxic circumstances [107]. These influences can decrease the ability of tissues to scavenge the produced free radicals. Additionally, AFB1 has been stated to diminish the absorption of the vitamins, inhibiting the body’s anti-defense mechanism [109].

The main route of AFB1 detoxification is through GST enzymes that conjugate AFBO with GSH. The basic determinant of species sensitivity to AFB1 is the efficiency and rate of GST activity [110]. Cellular GSH is an important regulator of some biological processes, including the proteins and DNA synthesis, affecting cell proliferation and growth, immunity, apoptosis, amino acid transport, endogenous and xenobiotic oxidant detoxification/metabolism, redox-sensitive signal transduction, etc. [18,111]. Contrarily, the GSH thiolic group can directly react with and detoxify a range of ROS, including superoxide anion, hydroperoxides H_2_O_2_, alkoxyl radicals, and hydroxyl radicals [112]; there is also a range of proteins with GSH dependent on hydroperoxides activity, including GSH-px, peroxiredoxins (Prx)-isoforms, glutathione reductase (Grx), and many GST [113]. 

## 5. Conclusions

*Arctostaphylos uva-ursi* extract, when injected in ovo, modulates the adverse effects of AFB1 in broiler embryos contaminated with AFB1, as it improves oxidative stress biomarkers, liver and kidney function, and immunological responses in broiler chicks. In addition, histological data revealed that *Ar. uu.*, reduced the embryotoxicity induced by 50 ng AFB1/10 µL. As a result, *Ar. uu.*, reduced the antioxidant enzymes’ capacity to counteract oxidative damage generated by AFB1. The recent findings will add to our understanding of the 0.01 g *Ar. uu.*/500 µL of *Ar. uu.*, in ovo injection as a therapeutic agent against AFB1-induced embryotoxicity.

## Figures and Tables

**Table 1:** Total phenolic content (TPC), total flavonoid content (TFC), and antioxidant activity of *Arctostaphylos uva-ursi*.

Methanol Extract	TPC mg GAE/g DW	TFC mg QE/g DW	Antioxidant Activity (DPPH %)
*Arctostaphylos uva-ursi*	212.88	91.28	60.25

**Table 2:** Bioactive compounds detected from methanolic extract of *Arctostaphylos uva-ursi*.

RT * (Min)	Compound Name	Structure	Compound Nature	Peak Area	Peak Area (%)
10.295	Thymine	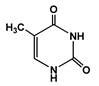	Alkaloids	109,350,462.3	17.40
11.323	4H-Pyran-4-one, 2,3-dihydro-3,5-dihydroxy-6-methyl-	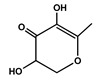	Phenols	153,643,563.1	24.45
12.171	Phloroglucinol, trimethylsilyl ether	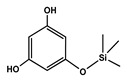	Phenols	3,447,892.805	0.55
12.407	Benzofuran, 2,3-dihydro-	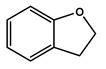	Coumarins	71,308,776.28	11.34
12.727	5-Hydroxymethylfurfural	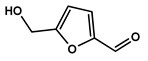	Glycosides	275,409,104.1	43.83
13.093	1,2-Benzenediol, 3-methoxy-	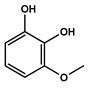	Phenols	3,877,902.97	0.62
13.748	2-Methoxy-4-vinylphenol	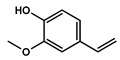	phenols	4,725,079.814	0.75
14.222	trans-3-Trifluoromethylcinnamic acid, 4-nitrophenyl ester	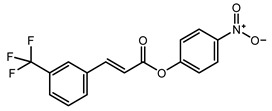	Cinnamic derivatives	134,564.4497	0.021
15.784	Succinic acid, 3-nitrobenzyl pentyl ester	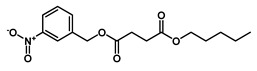	Organic acids	191,184.1509	0.030
16.270	Phenol, 2,6-bis(1,1-dimethylethyl)-	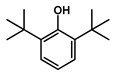	Phenols	777,540.8755	0.12
21.138	n-Hexadecanoic acid	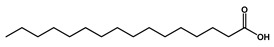	Long-chain fatty acid	2,705,961.767	0.43
22.821	9-Octadecenoic acid, (E)-	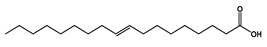	Unsaturated fatty acid	2,720,133.219	0.42

* Retention time (Min.).

**Table 3:** Liver and kidney functions of new hatched Ross broilers chicks (*n* = 10) exposed to AFB1 and/or *Ar. uu.* during incubation.

Items	Treatments						SEM	*p*-Value
	Control	DDW	Methanol	*Ar. uu.*	AFB1	AFB1 + *Ar. uu*		
Total Protein (g/L)	6.838 ^a^	6.806 ^a^	6.166 ^a^	6.527 ^a^	5.687 ^b^	6.398 ^a^	0.196	0.04
Albumin (g/L)	2.515 ^b^	2.631 ^b^	2.357 ^b^	2.210 ^b^	3.950 ^a^	2.833 ^b^	0.313	0.01
Globulin (g/L)	4.323 ^a^	4.175 ^a^	3.809 ^a^	4.317 ^a^	1.737 ^b^	3.565 ^a^	0.270	0.01
Alb/Glo ratio	0.582 ^b^	0.630 ^b^	0.619 ^b^	0.512 ^b^	2.274 ^a^	0.795 ^b^	0.109	0.01
GGT (IU)	0.650 ^c^	0.670 ^c^	0.688 ^c^	0.676 ^c^	1.340 ^a^	0.836 ^b^	0.002	0.01
AKP (IU)	549.33 ^c^	524.33 ^c^	577.73 ^c^	538.67 ^c^	1576.00 ^a^	886.67 ^b^	27.72	0.01
GPT (IU)	22.33 ^c^	24.24 ^c^	25.18 ^c^	23.40 ^c^	35.93 ^a^	28.67 ^b^	1.118	0.01
GOT (IU)	2.513 ^c^	2.716 ^c^	2.889 ^c^	12.963 ^c^	6.856 ^a^	3.622 ^b^	0.019	0.01
Urea Nitrogen	1.22 ^c^	1.19 ^c^	1.22 ^c^	1.24 ^c^	2.15 ^a^	1.53 ^b^	0.004	0.01
Uric acid (µmol/L)	0.831 ^c^	0.809 ^c^	0.882 ^c^	0.848 ^c^	1.99 ^a^	0.457 ^b^	0.110	0.01
C K * (mmol/L)	1.100 ^c^	1.06 ^c^	1.09 ^c^	1.01 ^c^	2.03 ^a^	1.40 ^b^	0.004	0.01

^a—c^ Values within a row with different letters differ significantly (*p* < 0.05), Values are expressed as mean ± SEM (*n* = 15), * = Creatinine Kinase, Control = non-injected eggs, DDW = Eggs injected with 10 μL DDW, Methanol= eggs injected with 500 µL Methanol, *Ar. uu.* = Eggs injected with 500 µL of a freshly prepared solution of 0.01 g of *Ar. uu.*, AFB1 = eggs injected with 10 μL DDW containing 50 ng AFB1, and AFB1 + *Ar. uu.* = Eggs injected with 10 μL DDW containing 50 ng AFB1 + 500 µL containing 0.01 g *Ar. uu*.

**Table 4:** Antioxidant indices, activities of dehydrogenase enzymes (GluDH, LDH and SDH) in liver, kidneys, and heart tissues of newly hatched broilers chicks exposed to AFB1 or/and Bearberry during incubation. (*n* = 10).

Items	Treatment Groups						SEM	*p*-Value
	Control	DDW	Methanol	*AR. UU.*	AFB1	AFB1 + *Ar. uu.*		
**Serum**								
TAOC (U/mg prot.)	6.04 ^a^	6.24 ^a^	6.30 ^a^	5.66 ^b^	2.66 ^d^	4.67 ^c^	0.016	0.01
SOD (U/mg prot.)	102.33 ^b^	100.66 ^b^	102.14 ^b^	113.66 ^a^	76.00 ^d^	88.00 ^c^	5.503	0.01
GSH (µmol/g prot.)	13.21 ^b^	9.03 ^c^	6.73 ^c^	12.24 ^b^	8.75 ^c^	17.59 ^a^	1.187	0.01
GSH-Px activity (U)	958.1 ^bc^	917.6 ^bc^	937.0 ^bc^	2579.3 ^a^	621.6 ^c^	1306.1 ^b^	61.371	0.01
Catalase (U/mg prot.)	47.02 ^b^	44.78 ^b^	43.61 ^b^	54.79 ^a^	19.01 ^d^	34.76 ^c^	2.741	0.01
MDA (nmol/mg prot.)	7.93 ^c^	5.67 ^e^	3.96 ^e^	6.77 ^cd^	18.97 ^a^	10.29 ^b^	0.545	0.01
LDH (U/mL)	831.41 ^b^	947.54 ^b^	1026.94 ^b^	851.80 ^b^	1428.91 ^a^	1038.04 ^b^	25.365	0.01
SDH (U/mL)	21.66 ^e^	28.00 ^cd^	33.19 ^c^	24.66 ^ed^	81.00 ^a^	50.00 ^b^	2.019	0.01
GluDH (U/mL)	34.00 ^a^	31.66 ^a^	30.64 ^a^	33.00 ^a^	22.33 ^b^	33.00 ^a^	3.483	0.01
**Liver**								
TAOC (U/mg prot.)	256.06 ^a^	264.57 ^a^	260.80 ^a^	240.18 ^b^	112.99 ^d^	198.10 ^c^	3.138	0.01
SOD (U/mg prot.)	433.74 ^b^	426.67 ^b^	430.34 ^b^	481.78 ^a^	322.12 ^d^	372.99 ^c^	5.739	0.01
GSH (µmol/g prot.)	5.60 ^b^	4.83 ^c^	4.64 ^bc^	5.18 ^b^	3.71 ^c^	4.75 ^a^	0.248	0.01
GSH-Px activity (U)	406.11 ^bc^	388.94 ^bc^	403.43 ^bc^	458.93 ^b^	263.44 ^c^	330.58 ^b^	46.540	0.01
Catalase (U/mg prot.)	49.83 ^b^	47.45 ^b^	48.28 ^b^	58.05 ^a^	20.15 ^d^	36.84 ^c^	1.007	0.01
MDA (nmol/mg prot.)	4.20 ^c^	3.00 ^d^	3.45 ^cd^	3.58 ^cd^	10.05 ^a^	5.45 ^b^	0.214	0.01
LDH (U/mL)	1276.76 ^c^	1304.43 ^c^	1300.63 ^c^	1206.56 ^c^	2597.06 ^a^	1633.50 ^b^	27.900	0.01
SDH (U/mL)	11.47 ^d^	14.83 ^c^	13.54 ^cd^	13.06 ^cd^	42.91 ^a^	26.49 ^b^	0.650	0.01
GluDH (U/mL)	180.13 ^a^	177.77 ^a^	172.52 ^a^	174.83 ^a^	138.65 ^c^	154.83 ^b^	5.684	0.01
**Kidney**								
TAOC (U/mg prot.)	107.93 ^a^	111.51 ^a^	109.92 ^a^	101.23 ^b^	47.62 ^d^	83.50 ^c^	1.322	0.01
SOD (U/mg prot.)	182.82 ^b^	179.84 ^b^	181.39 ^b^	203.07 ^a^	135.77 ^d^	157.21 ^c^	2.419	0.01
GSH (µmol/g prot.)	2.36 ^a^	1.61 ^c^	1.96 ^bc^	2.18 ^b^	1.56 ^c^	2.14 ^b^	0.104	0.01
GSH-Px activity (U)	171.17 ^b^	163.94 ^bc^	170.04 ^b^	196.80 ^a^	111.04 ^d^	146.52 ^c^	19.616	0.01
Catalase (U/mg prot.)	21.00 ^b^	20.00 ^b^	20.35 ^b^	24.47 ^a^	8.49 ^d^	15.52 ^c^	0.424	0.01
MDA (nmol/mg prot.)	1.77 ^c^	1.26 ^d^	1.45 ^cd^	1.51 ^cd^	4.23 ^a^	2.29 ^b^	0.090	0.01
LDH (U/mL)	1251.22 ^c^	1278.34 ^c^	1274.62 ^c^	1245.12 ^c^	2682.43 ^a^	1661.25 ^b^	27.341	0.01
SDH (U/mL)	4.83 ^d^	6.25 ^c^	5.71 ^cd^	5.29 ^cd^	18.08 ^a^	11.16 ^b^	0.274	0.01
GluDH (U/mL)	75.92 ^a^	70.71 ^a^	72.71 ^a^	73.69 ^a^	49.87 ^b^	73.96 ^a^	2.396	0.01
**Heart**								
TAOC (U/mg prot.)	133.39 ^a^	137.82 ^a^	135.85 ^a^	125.11 ^b^	58.86 ^d^	103.19 ^c^	1.635	0.01
SOD (U/mg prot.)	225.94 ^b^	222.26 ^b^	224.18 ^b^	250.97 ^a^	167.80 ^d^	194.30 ^c^	2.989	0.01
GSH (µmol/g prot.)	2.91 ^b^	1.99 ^c^	2.42 ^bc^	2.70 ^b^	1.93 ^c^	3.88 ^a^	0.129	0.01
GSH-Px activity (U)	211.55 ^bc^	202.61 ^bc^	210.16 ^bc^	227.23 ^a^	137.23 ^c^	188.37 ^b^	24.24	0.01
Catalase (U/mg prot.)	25.95 ^b^	24.71 ^b^	25.15 ^b^	28.51 ^a^	10.34 ^d^	19.19 ^c^	0.524	0.01
MDA (nmol/mg prot.)	2.18 ^c^	1.56 ^d^	1.80 ^cd^	1.86 ^cd^	5.23 ^a^	2.84 ^b^	0.111	0.01
LDH (U/mL)	1280.63 ^c^	1308.39 ^c^	1304.57 ^c^	1210.21 ^c^	2604.93 ^a^	1638.44 ^b^	27.98	0.01
SDH (U/mL)	5.98 ^d^	7.72 ^c^	7.05 ^cd^	6.80 ^cd^	22.35 ^a^	13.80 ^b^	0.339	0.01
GluDH (U/mL)	93.83 ^a^	87.39 ^a^	89.87 ^a^	91.07 ^a^	61.63 ^b^	91.07 ^a^	2.96	0.01

^a–e^ Values within a row with different letters differ significantly (*p* < 0.05), Values are expressed as mean ± SEM (*n* = 15), Control = Non-injected eggs, DDW = Eggs injected with 10 μL DDW, Methanol = eggs injected with 500 µL Methanol, *Ar. uu.* = Eggs injected with 500 µL of a freshly prepared solution of 0.01 g of *Ar. uu.*, AFB1 = eggs injected with 10 μL DDW containing 50 ng AFB1, and AFB1 + *Ar. uu.* = Eggs injected with 10 μL DDW containing 50 ng AFB1 + 500 µL containing 0.01 g *Ar. uu*.

## Data Availability

The data presented in this study are available upon request from the corresponding author.
